# Virulence factors and antimicrobial resistance profiles of *Enterococcus faecalis* and *Enterococcus faecium* isolates from urine samples of dogs and cats

**DOI:** 10.4142/jvs.26007

**Published:** 2026-07-06

**Authors:** Dawid Jańczak, Piotr Górecki, Jakub Kędziorek, Mateusz Antecki, Olga Szaluś-Jordanow

**Affiliations:** 1 Department of Infectious and Invasive Diseases and Veterinary Administration, Institute of Veterinary Medicine, Faculty of Biological and Veterinary Sciences, Nicolaus Copernicus University, 87-100 Toruń, Poland.; 2 Animallab Veterinary Laboratory, 03-430 Warsaw, Poland.; 3 Department of Small Animal Diseases with Clinic, Institute of Veterinary Medicine, Warsaw University of Life Sciences-SGGW, 02-776 Warsaw, Poland.

**Keywords:** *Enterococcus*, urinary tract infections, virulence, multidrug resistance, nitrofurantoin

## Abstract

**Importance:**

Enterococci are opportunistic uropathogens increasingly associated with recurrent urinary tract infections (UTIs) in companion animals, raising concerns about their virulence potential and high antimicrobial resistance.

**Objective:**

To characterize the species distribution, virulence-associated genes, and antimicrobial resistance profiles of urinary *Enterococcus* isolates from dogs and cats with recurrent UTIs, including vancomycin resistance determinants.

**Methods:**

This study analyzed 106 monomicrobial urinary *Enterococcus* isolates from 61 dogs and 45 cats with recurrent UTIs. Species identification was followed by multiplex polymerase chain reaction for *vanA*, *vanB*, *vanC1*, and *vanC2* and for virulence-associated genes (*fsr*, *efm*, *esp*, *cylA*, *cad1*, *ace, gelE*, and *asa1*). The antimicrobial susceptibility was assessed by disc diffusion. The associations were evaluated using univariate comparisons and multivariable analyses.

**Results:**

Among the 106 isolates, 68 (64.2%) were *Enterococcus faecalis* and 38 (35.8%) *Enterococcus faecium*. *E. faecalis* was more frequently isolated from cats than dogs (77.8% vs. 54.1%; odds ratio, 2.97). The virulence profiles differed according to species; *E. faecalis* was more likely to carry *ace*, *gelE*, *asa1*, *cylA*, *fsr*, and *esp*, and showed a higher overall virulence profile. The prevalence of resistance was high for erythromycin (82.1%), rifampicin (78.3%), tetracyclines (70.8%), and fluoroquinolones (~59%), with multidrug resistance detected in 84.9% of isolates. Nitrofurantoin resistance was more common in *E. faecium* than *E. faecalis* (47.4% vs. 5.9%) and was independently associated with *E. faecium* in multivariable analyses. The vancomycin-resistance gene *vanB* was detected in 2.83% of isolates.

**Conclusions and Relevance:**

Recurrent UTIs in dogs and cats were associated with high rates of multidrug-resistant *Enterococcus* isolates and species-specific virulence and resistance patterns. These findings support the use of species-level identification and susceptibility testing–guided therapy to improve antibiotic stewardship and the clinical outcomes in recurrent UTIs in companion animals.

## INTRODUCTION

Bacterial urinary tract infections (UTIs) are a common reason for veterinary consultations and a major driver of antimicrobial prescribing in dogs and cats. Current International Society for Companion Animal Infectious Diseases (ISCAID) guidance emphasizes culture-based diagnosis and distinguishes sporadic from recurrent infections and related syndromes, such as pyelonephritis, prostatitis, and, in some cases, subclinical bacteriuria, because these entities differ in prognosis, underlying contributors, and antimicrobial indications [1]. In dogs, recurrent UTI is a well-recognized clinical problem and is defined as two or more episodes within 12 months. The condition is often considered complicated because recurrence typically reflects persistent predisposing factors rather than sporadic exposure [2]. In cats, bacterial UTI accounts for a smaller proportion of feline lower urinary tract disease (FLUTD), but becomes more relevant with increasing age and comorbidity, where positive cultures may occur with or without overt lower urinary tract signs [3].

Although Enterobacterales, especially *Escherichia coli*, remain the most frequent uropathogens, a range of other organisms, including *Proteus* spp., staphylococci, streptococci, enterococci, and non-fermenting rods, contribute to the uropathogen burden depending on the clinical population, sampling method, and prior antimicrobial exposure [4,5]. Importantly, *Enterococcus* spp. represent a meaningful component of canine and feline urine bacteriology and may be disproportionately represented in polymicrobial cultures, consistent with their opportunistic ecology and association with complicated urinary tract disease and impaired urogenital defenses [5]. As typical gastrointestinal commensals that are environmentally resilient, enterococci can colonize multiple niches and may be of equivocal significance if interpreted without clinical context [6,7]. In dogs, urinary enterococci are frequently associated with recognized complicating factors, such as catheterization, urolithiasis, endocrinopathies, anatomic abnormalities, neurologic dysfunction, or immunomodulatory therapy. The case-control data suggest that *Enterococcus* spp. may be more associated with recurrent bacteriuria than *E. coli*, supporting their relevance in recurrent disease settings [7].

From a pathobiological perspective, persistence in the urinary tract and the capacity to acquire and disseminate antimicrobial resistance (AMR) determinants are the key features of urinary enterococci. Experimental and clinical observations support the importance of adherence and surface-associated phenotypes under relevant host conditions [8]. *Enterococcus faecalis* and *Enterococcus faecium* can harbor virulence-associated loci implicated in adherence and biofilm-related persistence, including enterococcal surface protein (*esp*), aggregation substance (*asa1*), gelatinase (*gelE*), and cytolysin/hemolysin-associated factors such as *cylA*. The *fsr* regulatory system is linked mechanistically to *E. faecalis* biofilm formation and regulates the secretion of *gelE* and related proteases [9,10,11]. Collagen-binding adhesins, such as *ace*, may also support colonization through Microbial Surface Components Recognizing Adhesive Matrix Molecules-mediated interactions with the host extracellular matrix components [6]. AMR in *Enterococcus* spp., which exhibit intrinsic reduced susceptibility to multiple agents and readily acquire resistance via horizontal transfer of mobile genetic elements, is a parallel One Health concern [6]. Surveillance data consistently indicate broader resistance in *E. faecium* than *E. faecalis*, with implications for empiric therapy and treatment failure [12]. Transferable glycopeptide resistance determinants are of particular concern in *E. faecium*. Although glycopeptides are not used for routine small-animal UTI therapy, the urinary tract may serve as a reservoir for multidrug-resistant (MDR) enterococci and resistance determinants under selective pressures such as prior antimicrobial exposure and recurrent disease [1,6,7,12].

Accordingly, 106 urinary *Enterococcus* spp. isolates from dogs and cats with recurrent UTIs were characterized for selected virulence-associated genes (*fsr*, *efm*, *esp*, *cylA*, *cad1*, *ace*, *gelE*, *asa1*), glycopeptide resistance determinants (*vanA*, *vanB*, *vanC1*, and *vanC2*), and phenotypic susceptibility to a clinically relevant antimicrobial panel. These data were integrated at the species level (*E. faecalis* vs. *E. faecium* and dog vs. cat) to support diagnostic investigations, targeted treatments, and antimicrobial stewardship in recurrent urinary tract disease in companion animals.

## METHODS

### Bacterial isolation and culture conditions

Urine samples from dogs and cats with recurrent UTIs were submitted for routine bacteriological diagnostics. Primary isolation was performed on solid media, using Enterococcosel Agar, Columbia Agar, and MacConkey Agar (GRASO Biotech, Starogard Gdański, Poland). The plates were incubated aerobically at 37°C for 24–48 h. Presumptive enterococcal colonies were subcultured to obtain pure isolates for further analyses. The eligible cases were restricted to canine and feline urine samples in which a bacteriological culture yielded a monomicrobial growth of *Enterococcus* spp.

### DNA extraction

The genomic DNA was extracted from freshly grown pure cultures using the Sherlock DNA extraction kit (AA Biotechnology, Gdańsk, Poland) according to the manufacturer’s instructions. The extracted DNA was stored at −20°C for further testing.

### Polymerase chain reaction (PCR) procedures for species identification, vancomycin resistance, and virulence profiling

Species-level identification of *Enterococcus* spp. (including *E. faecalis* and *E. faecium*), detection of glycopeptide resistance determinants (*vanA*, *vanB*, *vanC1*, and *vanC2*), and virulence genotyping (*fsr*, *efm*, *esp*, *cylA*, *cad1*, *ace*, *gelE*, and *asa1*) were performed using previously published multiplex PCR protocols [13,14,15] with the primer sets listed in Supplementary Tables 1, 2, 3. Unless stated otherwise, the reactions were evaluated using StartWarm HS-PCR Mix (A&A Biotechnology, Gdańsk, Poland) with 3 µL of template DNA and ultrapure water to a final volume. Each primer (10 µM) was added in a 1 µL per reaction. For the *Enterococcus* spp. virulence panel, the reactions were run in 50 µL (25 µL HS-PCR Mix), whereas species identification and vancomycin resistance screening were performed in 25 µL reactions (12.5 µL HS-PCR Mix). Thermal cycling consisted of 35 cycles with denaturation at 95°C for 30 sec, annealing at 55°C for 60 sec (or 54°C for the vancomycin resistance assay), and extension at 72°C for 60 sec. All PCRs were carried out on a MultiGene OptiMax thermocycler (Labnet International, Taoyuan, Taiwan). The amplicons were separated by electrophoresis on 1.5% agarose gel and visualized under UV illumination.

### Antimicrobial susceptibility testing (AST)

Phenotypic AST was conducted using the disc diffusion method. The inocula were prepared from fresh cultures and plated onto Mueller–Hinton II Agar, and incubated at 35°C for 24 h. Zone diameter interpretation was based primarily on veterinary interpretive criteria from Clinical & Laboratory Standards Institute (CLSI) VET01S and related veterinary standards [16,17]. Interpretive criteria were applied according to CLSI M100 when veterinary-specific breakpoints were unavailable for a given antimicrobial-organism combination [18]. For florfenicol, the breakpoints were applied using those established for chloramphenicol because the florfenicol-specific breakpoints were unavailable [19]. Similarly, the breakpoints for marbofloxacin and enrofloxacin were implemented based on those validated for *Staphylococcus*, consistent with previous publications [20]. For antimicrobial–organism combinations where an intermediate category was not defined in the referenced criteria, the isolates were categorized as S or R only, and ‘I’ was recorded as not applicable. The antimicrobial panel included the following: penicillin (PEN), ampicillin (AMP), florfenicol (FFC), chloramphenicol (CHL), ciprofloxacin (CIP), enrofloxacin (ENR), marbofloxacin (MAR), erythromycin (ERY), rifampicin (RIF), nitrofurantoin (NIT), linezolid (LZD), doxycycline (DOX), and tetracycline (TET), gentamicin (GEN) and streptomycin (STR) (Supplementary Table 4).

### Statistical analysis

All statistics were calculated on R (R Foundation for Statistical Computing, Vienna, Austria; version 4.x) using two-tailed tests with a *p* value < 0.05 threshold for statistical importance. A Fisher’s exact test was used for comparisons of categorical variables. The effect sizes were reported as odds ratios (OR) with 95% confidence intervals (CIs). The median and interquartile range (IQR) were used to describe the continuous variables, such as age. The Mann–Whitney *U* test was used to compare those variables. Multiple comparisons within panels (virulence genes and antimicrobial agents) were controlled by adjusting the *p* values using the Benjamini–Hochberg false discovery rate (FDR) procedure and are reported as *q* values. Multivariable associations with selected resistance outcomes were estimated using binary logistic regression models including *Enterococcus* species (*E. faecium* vs. *E. faecalis*), host species (cat vs. dog), and age (per 1-year increase) as predictors. The results are the adjusted OR (95% CI). All analyses were performed using R. Fisher’s exact test, the Mann–Whitney *U* test, and logistic regression where appropriate. The ORs with 95% CIs were calculated, and a false discovery rate correction was applied using the Benjamini–Hochberg procedure.

## RESULTS

### Isolates and host characteristics

**Fig. 1:**
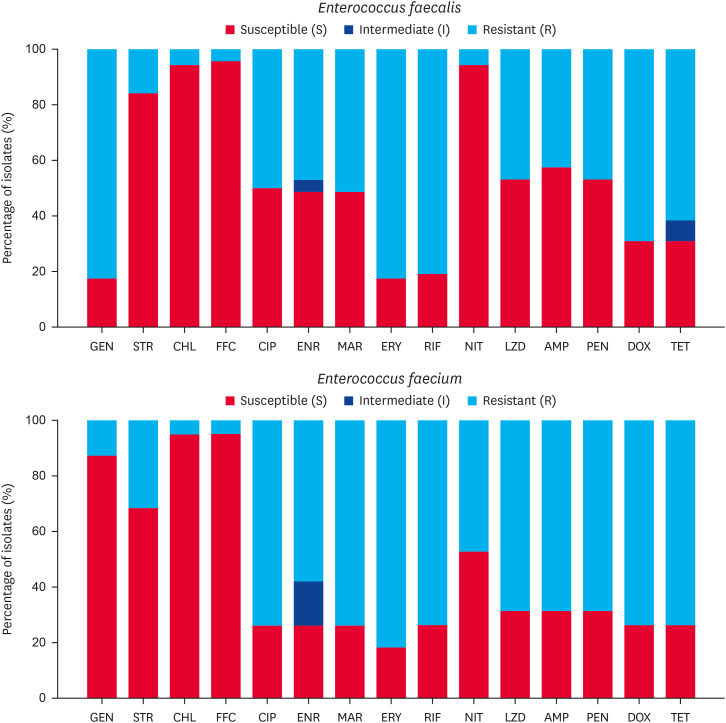
Percentage distribution of urinary enterococcal isolates classified as susceptible (S), intermediate (I), or resistant (R) to the tested antimicrobials (disk diffusion). PEN, penicillin; AMP, ampicillin; FFC, florfenicol; CHL, chloramphenicol; CIP, ciprofloxacin; ENR, enrofloxacin; MAR, marbofloxacin; ERY, erythromycin; RIF, rifampicin; NIT, nitrofurantoin; LZD, linezolid; DOX, doxycycline; TET, tetracycline; GEN, gentamicin; STR, streptomycin.

The isolates included urinary *Enterococcus* spp. isolates from 61 dogs (57.5%) and 45 cats (42.5%). Of the 106 isolates, 68 (64.2%) were *E. faecalis* and 38 (35.8%) were *E. faecium* (Fig. 1). The cats were far more likely than dogs to be positive for *E. faecalis*, at 77.8% vs. 54.1% (OR, 2.97; 95% CI, 1.25–7.05; Fisher’s exact *p* = 0.014). The median age of the animals was 8 versus 7 years (Mann-Whitney *p* = 0.513). Animals infected with *E. faecalis* tended to be older than those infected with *E. faecium* (median age 8 vs. 5.75, respectively), but this difference was not statistically significant (Mann–Whitney *p* = 0.065) (Table 1).

**Table 1:** Host characteristics and distribution of *Enterococcus* species among urinary isolates (n = 106)

Variable	n (%) or median (IQR)		OR [95% CI]	*p* value
	Dogs (n=61)	Cats (n=45)	Effect size (cats vs. dogs)	
Isolates	61 (57.5%)	45 (42.5%)	—	—
Female	47 (77.0%)	31 (68.9%)	0.66 [0.28–1.57]	0.379
Male	14 (23.0%)	14 (31.1%)	1.52 [0.64–3.61]	-
Age (yr)	8.00 (5.00–11.00)	7.00 (4.00–12.00)	—	0.513
*E. faecalis*	33 (54.1%)	35 (77.8%)	2.97 [1.25–7.05]	0.0144^*^
*E. faecium*	28 (45.9%)	10 (22.2%)	0.34 [0.14–0.80]	—

Fisher’s exact test for categorical variables; Mann–Whitney *U* test for age. The effect size was reported only for selected binary outcomes and for complementary categories; values are omitted as redundant (—).

IQR, interquartile range; OR, odds ratio; CI, confidence interval.

Asterisks indicate statistical significance: ^*^p < 0.05.

### Virulence gene profiles

The distribution of virulence genes in *E. faecalis* and *E. faecium* was significantly different. The most common genes were *cad1* (67.9%), *ace* (61.3%), and *gelE* (58.5%). The *ace* occurrence was significantly higher in *E. faecalis* (94.1% vs. 2.6%, OR, 592; *p* = 7.46 × 10^−23^; FDR-*q* = 5.97×10^−22^). The following genes were more prevalent in *E. faecalis* than in *E. faecium: gelE* (73.5% vs. 31.6%, OR, 6.02; *p* = 3.53 × 10^−5^; *q* = 5.65 × 10^−5^), *asa1* (54.4% vs. 7.9%, OR, 13.9; *p* = 9.28 × 10^−7^; *q* = 2.47 × 10^−6^), and *cylA* (29.4% vs. 2.6%, OR, 15.4; *p* = 6.60 × 10^−4^; *q* = 8.80 × 10^−4^). In contrast, *efm* appeared strongly associated with *E. faecium* (63.2% vs. 0.0%, OR, 0.00432). The cumulative virulence gene score (0–8 markers present) was significantly higher for *E. faecalis* than *E. faecium* (median 4 vs. 2, Mann–Whitney *p* = 3.34 × 10^−13^) (Table 2).

**Table 2:** Distribution of the virulence genes by *Enterococcus* species

Gene	n/N (%)		OR [95% CI]	*p* value	*q*
	*E. faecalis*	*E. faecium*	OR (*faecalis* vs. *faecium*)		
*fsr*	21/68 (30.9%)	0/38 (0.0%)	34.85 [2.04–594.11]	2.67E-05	5.35E-05^***^
*efm*	0/68 (0.0%)	24/38 (63.2%)	0.00432 [0.00025–0.07519]	2.49E-14	9.95E-14^***^
*esp*	18/68 (26.5%)	2/38 (5.3%)	6.48 [1.41–29.70]	0.00864	0.00987^**^
*cylA*	20/68 (29.4%)	1/38 (2.6%)	15.42 [1.98–120.19]	0.00066	0.00088^***^
*cad1*	50/68 (73.5%)	22/38 (57.9%)	2.02 [0.87–4.68]	0.129	0.129
*ace*	64/68 (94.1%)	1/38 (2.6%)	592.00 [63.76–5,496.82]	7.46E-23	5.97E-22^***^
*gelE*	50/68 (73.5%)	12/38 (31.6%)	6.02 [2.52–14.38]	3.53E-05	5.65E-05^***^
*asa1*	37/68 (54.4%)	3/38 (7.9%)	13.92 [3.90–49.68]	9.28E-07	2.47E-06^***^

Comparisons were performed using Fisher’s exact test. ORs are shown for *E. faecalis* versus *E. faecium*. Multiple-comparison adjustment was performed using the Benjamini–Hochberg FDR method and is reported as *q*. The *p* values are two-tailed.

n, gene-positive isolates; N, isolates tested within the corresponding species group; OR, odds ratio; CI, confidence interval.

Asterisks indicate statistical significance: ^**^q < 0.01, ^***^q < 0.001.

### Vancomycin-resistance genotypes

Vancomycin-resistance determinants were rare; *vanB* was detected in 3 *E. faecium* isolates, whereas *vanA*, *vanC1*, and *vanC2* were not detected.

### Antimicrobial resistance patterns and MDR

Across all isolates, phenotypic resistance was most frequent to erythromycin (87/106; 82.1%), rifampicin (83/106; 78.3%), doxycycline (75/106; 70.8%), tetracycline (70/106; 66.0%), marbofloxacin (63/106; 59.4%), ciprofloxacin (62/106; 58.5%), and enrofloxacin (54/106; 50.9%). Nine isolates (9/106; 8.5%) were intermediate to enrofloxacin, and five (5/106; 4.7%) were intermediate to tetracycline (Fig. 1). Gentamicin resistance was rare (5/106; 4.7%). Nitrofurantoin resistance differed by species: 4/68 (5.9%) in *E. faecalis* and 18/38 (47.4%) in *E. faecium* (OR, 0.07; *p* = 9.49 × 10^−7^; FDR-*q* = 1.42 × 10^−5^). After excluding IMP, 84/106 (79.2%) isolates were MDR: 52/68 (76.5%) *E. faecalis* and 32/38 (84.2%) *E. faecium* (OR, 0.61; 95% CI, 0.22–1.72; *p* = 0.456). The previous dog-versus-cat MDR comparison should be deleted (Table 3, Supplementary Table 5).

**Table 3:** Phenotypic antimicrobial resistance by *Enterococcus* species

Antibiotic	n/N (%)		OR [95% CI]	*p* value	*q*
	*E. faecalis*	*E. faecium*	OR (*faecalis* vs. *faecium*)		
NIT	4/68 (5.9%)	18/38 (47.4%)	0.07 [0.02–0.23]	9.49E-07	1.42E-05^***^
GEN	0/68 (0.0%)	5/38 (13.2%)	0.04 [0.00–0.83]	0.00495	0.0371^*^
AMP	29/68 (42.6%)	26/38 (68.4%)	0.34 [0.15–0.79]	0.0149	0.0743
CIP	34/68 (50.0%)	28/38 (73.7%)	0.36 [0.15–0.85]	0.0236	0.0886
ENR	32/68 (47.1%)	22/38 (57.9%)	0.65 [0.29–1.44]	0.316	0.474
MAR	35/68 (51.5%)	28/38 (73.7%)	0.38 [0.16–0.90]	0.0385	0.0913
LZD	32/68 (47.1%)	26/38 (68.4%)	0.41 [0.18–0.94]	0.0426	0.0913
PEN	32/68 (47.1%)	26/38 (68.4%)	0.41 [0.18–0.94]	0.0426	0.0913
STR	11/68 (16.2%)	12/38 (31.6%)	0.42 [0.16–1.07]	0.0859	0.161
RIF	55/68 (80.9%)	28/38 (73.7%)	1.51 [0.59–3.87]	0.463	0.632
DOX	47/68 (69.1%)	28/38 (73.7%)	0.80 [0.33–1.94]	0.663	0.828
TET	42/68 (61.8%)	28/38 (73.7%)	0.58 [0.24–1.38]	0.286	0.474
CHL	4/68 (5.9%)	2/38 (5.3%)	1.12 [0.20–6.45]	1	1
FFC	3/68 (4.4%)	2/38 (5.3%)	0.83 [0.13–5.20]	1	1
ERY	56/68 (82.4%)	31/38 (81.6%)	1.05 [0.38–2.95]	1	1

n, gene-positive isolates; N, isolates tested within the corresponding species group; OR, odds ratio; CI, confidence interval; NIT, nitrofurantoin; GEN, gentamicin; AMP, ampicillin; CIP, ciprofloxacin; ENR, enrofloxacin; MAR, marbofloxacin; LZD, linezolid; PEN, penicillin; STR, streptomycin; RIF, rifampicin; DOX, doxycycline; TET, tetracycline; CHL, chloramphenicol; FFC, florfenicol; ERY, erythromycin; FDR, false discovery rate.

Comparisons were performed using a Fisher’s exact test. ORs are shown for *E. faecalis* versus *E. faecium*. Multiple-comparison adjustment was performed using the Benjamini–Hochberg FDR method and is reported as *q*. The *p* values are two-tailed.

Asterisks indicate statistical significance: ^*^q < 0.05, ^***^q < 0.001.

### Multivariable predictors of selected resistance outcomes

The adjusted logistic regression models including *Enterococcus* species, host species, and age showed that *E. faecium* was independently associated with resistance to nitrofurantoin (adjusted OR, 26.24; 95% CI, 6.23–110.56; *p* = 8 × 10^−6^). *E. faecium* was also independently associated with fluoroquinolone-class resistance (OR, 2.71; 95% CI, 1.09–6.71; *p* = 0.0316), linezolid resistance (OR, 2.40; 95% CI, 1.00–5.76; *p* = 0.0497), and ampicillin resistance (OR, 2.89; 95% CI, 1.20–6.97; *p* = 0.0181). These models were also fitted to the host species and host age, neither of which was significantly associated with the evaluated resistance outcomes (Table 4).

**Table 4:** Multivariable logistic regression for selected resistance outcomes.

Resistance outcome	Predictor	OR (95% CI)	*p* value
Nitrofurantoin	*E. faecium* vs. *E. faecalis*	26.24 (6.23–110.56)	8.00E-06^***^
	Cat vs. Dog	3.54 (0.96–13.01)	0.0571
	Age (per 1 year)	1.06 (0.93–1.21)	0.397
Fluoroquinolone class	*E. faecium* vs. *E. faecalis*	2.71 (1.09–6.71)	0.0316^*^
	Cat vs. Dog	0.95 (0.42–2.17)	0.905
	Age (per 1 year)	1.02 (0.93–1.12)	0.646
Linezolid	*E. faecium* vs. *E. faecalis*	2.40 (1.00–5.76)	0.0497^*^
	Cat vs. Dog	1.10 (0.49–2.50)	0.815
	Age (per 1 year)	0.97 (0.89–1.07)	0.579
Ampicillin	*E. faecium* vs. *E. faecalis*	2.89 (1.20–6.97)	0.0181^*^
	Cat vs. Dog	1.20 (0.53–2.76)	0.662
	Age (per 1 year)	0.96 (0.88–1.06)	0.416

Adjusted ORs were obtained from multivariable logistic regression models. The *p* values are shown.

OR, odds ratio; CI, confidence interval.

Asterisks indicate statistical significance: ^*^p < 0.05, ^***^p < 0.001.

## DISCUSSION

Enterococci are opportunistic urinary pathogens in dogs and cats, and their clinical relevance depends on the disease phenotype, urine collection method, and comorbidities. International small-animal UTI guidelines emphasize that recurrent infections usually reflect persistent predisposing factors and should be managed with culture-based decisions and antimicrobial stewardship [1]. Selection for persistence-adapted lineages under host and antimicrobial pressure cannot be ruled out, given that the present dataset comprised recurrent, monomicrobial Enterococcus spp. isolates obtained from animals with recurrent UTIs. Nevertheless, the predominance of *E. faecalis* (64.2%) over *E. faecium* (35.8%) aligns with the clinical microbiology literature, whereas *E. faecium* is often overrepresented among highly resistant lineages and more complex presentations [5,6]. Finally, these results support routine species-level reporting of *Enterococcus* in clinical practice because *E. faecalis* was more common in cats, and crude host-species comparisons may be confounded by species composition [5,12]. The detection of a given locus should be interpreted as genotypic potential rather than proof of phenotypic expression or *in vivo* pathogenic activity because virulence profiling in this study relied on multiplex PCR. Expression of virulence traits is context-dependent and may vary across host conditions and regulatory backgrounds.

In the present study, significant differences in the virulence-marker carriage were observed between the two *Enterococcus* species. Moreover, the observed direction of these differences was, in most cases, biologically plausible. *E. faecalis* had considerably more *ace*, *gelE*, *asa1*, *cylA*, *fsr*, and *esp* genes and exhibited a significantly higher cumulative marker score than *E. faecium*. In contrast, *efm* was associated with *E. faecium*, and its determinants are located within a single persistence phenotype: adherence, biofilm formation, and tissue persistence.

The high prevalence of the *ace* gene in *E. faecalis* in this study suggests that it may facilitate the binding of *E. faecalis* to collagen and other extracellular matrix proteins, which could lead to mucosal colonization and potential persistence [21]. Typically, adherence is an important component of enterococcal pathogenesis, including the urinary tract [8].

The *fsr* locus encodes a quorum-sensing system that controls extracellular protease gelatinase, which has been implicated in *E. faecalis* biofilm and persistence [11]. The higher prevalence of *gelE* in *E. faecalis* in this population suggests that *E. faecalis* strains circulating in recurrent UTIs may be selectively enriched for biofilm-forming or persistence-associated traits.

In recurrent UTIs, antibiotic exposure and chronic inflammation-mediated alterations of the uroepithelium may confer persistence-related traits, such as aggregation substance and cytolysin, to the recurrent pathogens and may enhance uropathogenic traits [9,14].

The strong association between *efm* and *E. faecium* is consistent with its use as a species-associated marker and its putative link to adherence and biofilm-related phenotypes [12].

A practical implication is that species identity provides an immediate, clinically interpretable context even before whole-genome characterization. In this cohort of recurrent infections, *E. faecalis* tended to harbor a broader repertoire of virulence-associated genes, whereas *E. faecium* showed a distinct marker profile but a comparatively more problematic antimicrobial resistance phenotype. This division of virulence-marker burden vs. resistance burden across settings supports the combination of species identification and AST for clinical decision-making rather than either one alone [5,6].

Resistance to classes of antimicrobials not recommended as a first-line in clinical practice was also observed, and the incidence of MDR isolates was unexpectedly high (79.2%). This appears biologically plausible as recurrence is associated with previous antimicrobial exposure and antimicrobial stewardship guidelines recommend against using broad-spectrum agents as empiric treatment in all recurrent or complicated UTI cases [1,2].

The MDR prevalence observed in the present recurrent-UTI cohort (79.2%) is at the upper end of published veterinary reports. For example, urinary enterococci from dogs and cats in northwestern Croatia were reported to be predominantly MDR (> 75%) [22]. Large-scale surveillance of canine clinical enterococci in the northeastern United States also documented a substantial MDR burden and marked species-specific resistance profiles, particularly for *E. faecium* [12]. In contrast, studies focusing on enterococci from healthy companion animals may report substantially lower MDR levels and very low or absent linezolid non-susceptibility [23]. Collectively, these comparisons suggest that recurrent/complicated infection settings may function as high-selection niches, underscoring the need for culture and AST-guided therapy in recurrent UTIs.

The largest species-specific association was documented for a differential resistance pattern to nitrofurantoin, being low in *E. faecalis* (5.9%) and high in *E. faecium* (47.4%), with a parallel large effect size. Nitrofurantoin is generally considered a urinary agent because of its high concentrations in the urinary tract and high activity against some resistant uropathogens. Its clinical effectiveness depends in part on the susceptibility of the isolates and the infecting species. Recent veterinary pharmacokinetic studies suggest that recommended canine dosing can achieve urinary concentrations active against representative urinary pathogens, including E. faecium, but the clinical efficacy still depends on isolate-level susceptibility. This finding underscores the need for microbiological confirmation and clinical realism in nitrofurantoin use, rather than assuming that this high urinary drug concentrations necessarily ensure clinical efficacy [24]. The high rate of *E. faecium* resistance in this study supports rapid species identification and AST before definitive therapy for recurrent disease and argues against empirical nitrofurantoin use when *E. faecium* infection is suspected.

Multivariable logistic regression yielded similar results, with *E. faecium* remaining independently associated with resistance to nitrofurantoin, the fluoroquinolone class, linezolid, and ampicillin. Host species and animal age were not independently associated with any of the evaluated resistance outcomes. These findings support species-level identification and rapid AST so that urine-active agents of proven susceptibility are prioritized over inappropriate use of critically important antimicrobials [1].

A particularly concerning finding was the high proportion of linezolid non-susceptibility. Linezolid is an oxazolidinone that is considered a critically important agent in human medicine for infections caused by multidrug-resistant Gram-positive pathogens, including vancomycin-resistant Enterococcus (VRE). The contrast between the high linezolid resistance observed in the present study and reports showing very low or absent linezolid resistance in enterococci from healthy companion animals highlights the potential role of recurrent/complicated infection settings as selection niches [23]. Importantly, transferable determinants associated with oxazolidinone resistance, including optrA, poxtA, and cfr-like genes, have been detected in enterococci from pet-associated sources and can be linked to co-selection with phenicols [25,26]. Although these genes were not investigated in this study, the findings support confirmatory testing and targeted molecular surveillance for transferable oxazolidinone resistance determinants in clinical enterococci from companion animals.

Regarding glycopeptide resistance within a One Health framework, only *vanB* was detected in three *E. faecium* isolates (2.83%), whereas *vanA*, *vanC1*, and *vanC2* were not identified. Although glycopeptides are not used in routine small-animal UTI treatment, the van genes remain important within a One Health framework due to their potential for horizontal transfer and the prevalence of VRE lineages in human medicine [6,27].

Nevertheless, in recurrent infections associated with repeated antimicrobial exposure and environmental exchange, even infrequent detection of *van* genes in companion animals may be epidemiologically relevant [23,28].

These findings support the need for infection control measures and appropriate communication and surveillance related to antimicrobial stewardship. Recurrent UTI populations may act as “selection-and-maintenance” niches for enterococci, even among populations that may not have acute clinical need for glycopeptide therapy.

This study had several limitations. First, defining the cohort by recurrent UTIs with monomicrobial *Enterococcus* spp. culture may improve the interpretability at the expense of generalizability to the sporadic/uncomplicated cystitis setting or indeed to polymicrobial culture. Second, virulence profiling was limited to a predefined gene panel. Functional assays were not performed to assess biofilm formation, adhesion, or in vivo persistence, nor was the genomic context evaluated to define the genotype–phenotype relationships. Third, detailed clinical covariates, including urolithiasis, endocrinopathies, catheterization status, immunomodulatory therapy, and individual antimicrobial exposure history, were not consistently available. These unmeasured factors may influence the recurrence risk and resistance selection, and their absence limits causal inference regarding the associations observed in this retrospective laboratory-based dataset. The inclusion of these variables may improve the causal inference in future studies. The AST method and breakpoint selection could influence categorical interpretations, but the use of standardized methodologies and transparent interpretive criteria helps mitigate these limitations [16,17,18]. Moreover, for selected antimicrobial–organism combinations lacking veterinary *Enterococcus*-specific interpretive criteria, categorical S/I/R assignments were based on surrogate CLSI criteria. Therefore, the corresponding categorical proportions should be interpreted cautiously and primarily as descriptive comparisons within the cohort rather than as definitive clinical susceptibility categorizations. Linezolid resistance was assessed phenotypically by disk diffusion. Confirmatory minimum inhibitory concentration testing and molecular characterization of oxazolidinone resistance determinants were outside the scope of this study and should be addressed in follow-up studies.

Future studies should incorporate more comprehensive clinical covariates and whole-genome sequencing to characterize enterococcal virulence and antimicrobial resistance determinants in recurrent UTIs.

Clinical implications for recurrent canine and feline UTIs are as follows:

1. Recurrent UTIs should not be treated empirically for *Enterococcus* spp. infection. Cases of recurrent UTIs should be routinely cultured, and AST should be performed [1,2].
2. At the species level, *E. faecalis* was associated with a higher burden of virulence-associated markers, whereas *E. faecium* was associated with more restricted therapeutic options and broader antimicrobial resistance. These findings support routine species-level differentiation of urinary *E. faecalis* and *E. faecium* isolates.
3. Nitrofurantoin is not a universally reliable therapeutic option for *Enterococcus* spp. The high *E. faecium* resistance rate highlights the need for AST-guided use and caution in empirical selection [22,29].
4. Continued surveillance is warranted in settings where low-level *vanB* is detected. Rare *van* genes should be interpreted from a One Health perspective and integrated into surveillance, reporting, and stewardship programs [6,27,28].

## SUPPLEMENTARY MATERIALS

Supplementary Table 1 Oligonucleotide primer sequences and product lengths for species-level identification of *Enterococcus* spp.

Supplementary Table 2 Oligonucleotide primer sequences used for PCR detection of putative virulence genes in *Enterococcus* isolates

Supplementary Table 3 Oligonucleotide primer sequences used for PCR detection of vancomycin resistance determinants in *Enterococcus* isolates

Supplementary Table 4 Disk diffusion interpretive criteria and high-level aminoglycoside resistance screening thresholds used for *Enterococcus* spp.

Supplementary Table 5 Class-based resistance by *Enterococcus* species
